# Nanoprodrugs of NSAIDs: Preparation and Characterization of Flufenamic Acid Nanoprodrugs

**DOI:** 10.1155/2011/980720

**Published:** 2011-04-05

**Authors:** Bong-Seop Lee, Chi Woo Yoon, Arsen Osipov, Nuriel Moghavem, Daniel Nwachokor, Rina Amatya, Rebekah Na, Joe L. Pantoja, Michael D. Pham, Keith L. Black, John S. Yu

**Affiliations:** Department of Neurosurgery, Cedars-Sinai Medical Center, 8631 West Third Street, Suite 800 East, Los Angeles, CA 90048, USA

## Abstract

We demonstrated that hydrophobic derivatives of the nonsteroidal anti-inflammatory drug (NSAID)flufenamic acid (FA), can be formed into stable nanometer-sized prodrugs (nanoprodrugs) that inhibit the growth of glioma cells, suggesting their potential application as anticancer agent. We synthesized highly hydrophobic monomeric and dimeric prodrugs of FA via esterification and prepared nanoprodrugs using spontaneous emulsification mechanism. The nanoprodrugs were in the size range of 120 to 140 nm and physicochemically stable upon long-term storage as aqueous suspension, which is attributed to the strong hydrophobic interaction between prodrug molecules. Importantly, despite the highly hydrophobic nature and water insolubility, nanoprodrugs could be readily activated into the parent drug by porcine liver esterase, presenting a potential new strategy for novel NSAID prodrug design. The nanoprodrug inhibited the growth of U87-MG glioma cells with IC_50_ of 20 *μ*M, whereas FA showed IC_50_ of 100 *μ*M, suggesting that more efficient drug delivery was achieved with nanoprodrugs.

## 1. Introduction

Nonsteroidal anti-inflammatory drugs (NSAIDs) are a class of drugs with analgesic, antipyretic and anti-inflammatory effects and have been widely used in the treatment of pain, fever, and inflammation. NSAIDs exert their anti-inflammatory activity through the inhibition of cyclooxygenase (COX) derived prostaglandin synthesis. COX has been recognized as the first enzyme in the formation of prostaglandin (PG) and thromboxane (TX) from arachidonic acid at the site of inflammation or after infection [1]. COX-1 isozyme is expressed constitutively in many tissues, whereas COX-2 isozyme is expressed only at the site of inflammation [2]. Recent studies have conjectured that elevated expression of COX-2 has been detected in various cancers, including colorectal, lung, breast, liver, head and neck, and brain tumors, whereas COX-1 expression was unaffected [3–5]. Several studies have also demonstrated that NSAIDs may be effective in the prevention and treatment of certain types of cancers [6–9]. The chemopreventive and antitumorigenic effects of NSAIDs are believed to be exerted through the induction of apoptosis followed by inhibition of COX-2 [10–13]. Some data also suggest a COX-2-independent mechanism because apoptosis induction by NSAIDs does not always correlate with their ability to inhibit COX-2 [14–17].

However, the major mechanism by which NSAIDs exert their anti-inflammatory activity, the inhibition of cyclooxygenase-derived prostaglandin synthesis, is also responsible for the adverse side effects, such as irritation and ulceration of the gastrointestinal (GI) mucosa [18]. It is generally believed that these GI side effects result from the combined effect of the irritation caused by the free carboxylic groups in NSAIDs and blockage of prostaglandin biosynthesis in the GI tract [19]. 

Prodrug strategy is widely recognized as a potential approach to overcome toxic side effects that are ascribed to the irritation caused by the free carboxylic groups in NSAIDs and blockage of prostaglandin biosynthesis in the GI tract. There have been several attempts to develop prodrugs of NSAIDs to overcome the adverse side effects as well as to improve their bioavailability by masking the carboxylic acid groups through the formation of bioreversible bonds [20–24].

The development of nanostructured biomaterials with antitumorigenic efficacy has received significant attention from the pharmaceutical industry, mainly because of their potential for precise targeting with less severe toxic side effects. Many effective anticancer therapeutics are low water soluble and must be in excessive amounts of organic cosolvents to obtain a therapeutically effective dose. This limits clinical applicability of these drugs. The formation into compact nanostructures obviates the need to use organic solvents, eliminating the interference of toxic side effects caused by cosolvents [25, 26]. In addition, by using a nanometer-sized delivery system, a significant drug loading per unit volume can be achieved, which is of crucial importance when high dosing is required. 

**Figure 1:**
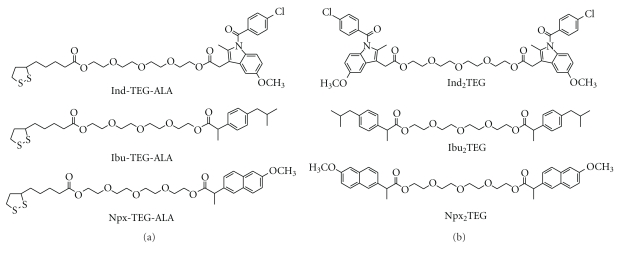
Nanoprodrugs of NSAIDs. ALA: *α*-lipoic acid; Ind: indomethacin; Ibu: ibuprofen; Npx: naproxen; TEG: tetraethylene glycol.

In our effort to combine the prodrug concept and nanostructured drug/drug delivery system we demonstrated that water-insoluble prodrug compounds can be transformed into stable nanostructures obviating the need to dissolve the compounds in organic solvents. In our previous report we demonstrated six hydrophobic derivatives of NSAIDs (Figure 1) and their nanoprodrugs [27, 28]. In this study, we synthesized monomeric and dimeric prodrugs of flufenamic acid (FA, 2-[(3-trifluoromethylphenyl)amino]benzoic acid) and prepared nanoprodrugs through spontaneous emulsification of the prodrugs in acetone. Further, we demonstrated the antiproliferative effect of FA nanoprodrugs on U87GM glioma cells.

## 2. Materials and Methods

### 2.1. General Procedures and Materials

Unless otherwise noted, solvents and chemicals were obtained at highest purity from Sigma-Aldrich Chemical Co. (St Louis, MO, USA) and used without further preparation. Chromatographic purification of the synthesized compounds was performed using silica gel (60 Å, 200–400 mesh). The compounds were confirmed by thin layer chromatography (TLC) silicagel plate (Merck 60 F254). Compounds containing *α*-lipoic acid were visualized by treatment with a solution of: 1.5 g of KMnO_4_, 10 g of K_2_CO_3_, and 1.25 mL of 10% NaOH in 200 mL of H_2_O, followed by gentle heating. The oxidized derivatives of FA were visualized under UV light. ^1^H and ^13^C NMR spectra were conducted on a Varian 400 MHz spectrometer and chemical shifts (*δ*) were given in ppm relative to TMS. The spectra were recorded with the solvent CDCl_3_ at room temperature.

### 2.2. High-Performance Liquid Chromatography

HPLC analysis was performed on a Merck-Hitachi analytical LaChrom D-7000 HPLC/UV detector system (Merck, Darmstadt, Germany) with a CAPCELL PAK, Type SG 120 (phenomenex, Torrance, CA, USA) C_18_ reversed phase column (250/4.6 mm, 5 *μ*m). The separation was performed under isocratic condition at a flow rate of 1 mL/min. The composition of the mobile phase (acetonitrile/water mixture containing 0.1% (v/v) trifluoroacetic acid) was adjusted for prodrugs and their degradation products in order to provide an appropriate retention time and separation. The detection was carried out at 254 nm.

### 2.3. Synthesis of FA Prodrugs

**Scheme 1:**
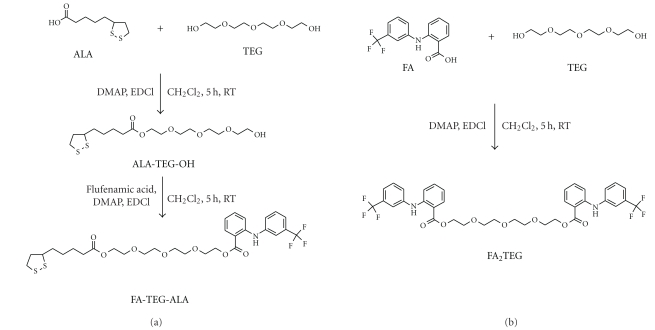
Synthesis of hydrophobic derivatives of FA.

The synthesis and characterization of the monomeric derivative of *α*-lipoic acid (ALA) with tetraethylene glycol (TEG) (ALA-TEG-OH, Scheme 1(a)) was performed as described in [27]. The synthesis and characterization of the monomeric (Scheme 1(a)) and the dimeric (Scheme 1(b)) FA derivatives were performed as follows.

ALA-TEG-OH (3.8 mmol) and FA (4.1 mmol, FA) in 20 mL of anhydrous dichloromethane (DCM) were reacted with 4-(dimethylamino)-pyridine (DMAP, 4.1 mmol) in the presence of molecular sieve for 10 min at room temperature. *N*-(3-Dimethylaminopropyl)-*N*-ethylcarbodiimide hydrochloride (EDCI, 4.1 mmol) was added portionwise over 10 min and the reaction mixture was stirred for 5 h at room temperature in the dark, filtered, and then concentrated under vacuum at room temperature. The products were purified using column chromatography (100 : 1 CH_3_Cl : MeOH) and characterized as described above (Section 2.1). 

 For the synthesis of dimeric derivative FA_2_TEG, FA (6 mmol), and TEG (2.5 mmol) in 40 mL of anhydrous DCM were reacted with DMAP (6 mmol) in the presence of molecular sieve for 10 min at room temperature. EDCI (6 mmol) was added portionwise over 10 min and the reaction mixture was stirred for 5 h at room temperature in the dark, filtered, and then concentrated under vacuum. The products were purified using column chromatography (100 : 0.5 CH_3_Cl : MeOH) and characterized as described above (Section 2.1). 

 FA-TEG-OH was synthesized and used for the identification of the degradation products of the monomeric and dimeric prodrugs during enzymatic hydrolysis. FA (10 mmol) and TEG (30 mmol) in 50 mL of anhydrous dichloromethane (DCM) were reacted with DMAP (15 mmol) in the presence of a molecular sieve (Fluka, 3 Å, 10–20 mesh beads) for 10 min at room temperature. EDCI (10 mmol) was added portionwise over 10 min and the reaction mixture was stirred for 5 h at room temperature in the dark, filtered, and then concentrated under vacuum to reduce the volume. The product FA-TEG-OH and dimeric byproduct FA_2_TEG were separated using column chromatography by loading the concentrated reaction mixture on the column without prior preparation and characterized as described above.

**FA-TEG-OH**

The column chromatography on silica gel (CHCl_3_ : MeOH 100 : 1) gave the compound as a colorless oil (75%). TLC (CHCl_3_ : MeOH 100 : 1) *R*_*f*_ 0.33; ^1^H NMR (400 MHz, CDCl_3_): *δ* = 3.51 (t, 2 × H), 3.60 (m, 10 × H), 3.82 (t, 2 × H), 4.42 (t, 2 × H), 6.80 (t, 1 × H), 7.18 (m, 2 × H), 7.40 (m, 4 × H), 8.05 (t, 1 × H), 9.53 (s, 1 × H). ^13^C NMR (100 MHz, CDCl_3_): *δ* = 61.66, 63.92, 69.15, 70.27, 70.52, 70.64, 70.68, 72.57, 113.02, 114.31, 117.93, 118.36, 119.49, 124.45, 129.90, 131.61, 131.99, 132.25, 134.31, 141.66, 146.63, 168.21.

**FA-TEG-ALA**

The column chromatography on silica gel (CHCl_3_ : MeOH 100 : 1) gave the compound as a yellow oil (65%). TLC (CHCl_3_ : MeOH 100 : 1) *R*_*f*_ 0.55; ^1^H NMR (400 MHz, CDCl_3_): *δ* = 1.49 (m, 2 × H), 1.70 (m, 4 × H), 1.90 (m, 1 × H), 2.45 (m, 1 × H), 3.10 (m, 2 × H), 3.59 (m, 1 × H), 3.70 (m, 10 × H), 3.82 (t, 2 × H), 4.20 (t, 2 × H), 4.45 (t, 2 × H), 6.80 (t, 1 × H), 7.25 (m, 2 × H), 7.35 (m, 4 × H), 8.07 (t, 1 × H), 9.52 (s, 1 × H). ^13^C NMR (100 MHz, CDCl_3_): *δ* = 24.61, 28.73, 33.94, 34.59, 38.48, 40.22, 56.34, 63.45, 63.96, 69.17, 70.56, 70.67, 70.75, 113.03, 114.33, 117.97, 118.08, 118.34, 119.52, 124.50, 129.11, 129.91, 131.98, 134.32, 141.69, 146.67, 168.22, 173.44.

**FA_2_TEG**

The column chromatography on silica gel (CHCl_3_ : MeOH 100 : 1) gave the compound as a colorless oil (75%). TLC (CHCl_3_ : MeOH 100 : 1) *R*_*f*_ 0.75; ^1^H NMR (400 MHz, CDCl_3_): *δ* = 3.65 (m, 8 × H), 3.85 (t, 4 × H), 4.45 (t, 4 × H), 6.80 (t, 1 × H), 7.20 (m, 2 × H), 7.40 (m, 4 × H), 8.00 (t, 1 × H), 9.53 (s, 2 × H). ^13^C NMR (100 MHz, CDCl_3_): *δ* = 63.95, 69.16, 70.72, 70.75, 113.02, 114.31, 117.96, 118.33, 119.51, 122.62, 125.33, 129.89, 131.67, 131.99, 134.31, 141.65, 146.67, 168.22.

### 2.4. Preparation of FA Nanoprodrugs

Nanoprodrugs were prepared according to the method using spontaneous emulsification as described [27]. Briefly, 25 mg of the FA derivatives and 5 mg of *α*-tocopherol were dissolved in acetone (5 mL) containing polysorbate 80 (0.1% w/v). The organic solution was poured under moderate stirring on a magnetic plate into an aqueous phase prepared by dissolving 25 mg of Pluronic F68 in 10 mL distilled water (0.25% w/v). Following 15 min of magnetic stirring, the acetone was removed under reduced pressure at room temperature. The suspensions were filtered through 0.8 *μ*m hydrophilic syringe filter (Corning, Part no 431221, Fisher Scientific Co., Pittsburgh, PA, USA), dialyzed in cellulose membrane tube (Sigma, code D9777) overnight in distilled water and stored at 4°C. A control nanosphere was prepared with 25 mg of *α*-tocopherol in the absence of FA derivatives using the same procedure as described above. To demonstrate cellular uptake, nanoprodrugs containing a hydrophobic fluorescent dye, coumarin 6 (Sigma, code 442631), were prepared using identical procedure except that 50 *μ*g of the dye was added to the organic FA prodrug solution prior to spontaneous emulsification. The incorporated dye remains associated with nanoprodrugs during dialysis overnight.

### 2.5. Size Measurements and Visualization of Nanoprodrugs

The hydrodynamic size measurement and size distribution of the nanoprodrugs were performed by the dynamic light scattering (DLS) using a Coulter N4-Plus Submicron Particle Sizer (Coulter Corporation, Miami, FL, USA) as described [27]. For each preparation mean diameter and mean polydispersity index (PI) of three determinations were calculated. The error bar (SD) was calculated from triplicate determinations. For visualization of the nanoprodrugs, nanoparticle tracking analysis (NTA) experiments were performed using a digital microscope LM10 System (NanoSight, Amesbury, UK). A small amount of the diluted nanoprodrug suspension in water was introduced into the chamber by a syringe. The particles in the sample were observed using the digital microscope. The movement of nanoprodrugs under Brownian motion was analyzed by the NTA, version 1.3 (B196) image analysis software (NanoSight).

### 2.6. Stability of FA Nanoprodrugs during Long-Term Storage

The stability of the nanoprodrugs was assessed by measuring the nanoprodrug size and concentrations of prodrug molecules after 8-week storage at 4°C. 

The size of the nanoprodrugs was measured as described above (Section 2.5). The amount of intact FA prodrugs was assessed by RP-HPLC as follows: the suspensions of nanoprodrugs (100 *μ*L) were added to acetonitrile (400 *μ*L) and analyzed using RP-HPLC as described (Section 2.2). The recovery yield was calculated as follows:

(1) $$\begin{array}{cc} & \text{Recovery}\;\text{yield}\;\left(\text{\%}\right)\\ & \;\;=\frac{\text{Amount}\;\text{of}\;\text{prodrugs}\;\text{after}\;\text{incubation}}{\text{Amount}\;\text{of}\;\text{prodrugs}\;\text{before}\;\text{incubation}}\times 100. \end{array}$$

The error bar (S.D.) was calculated from triplicate determinations.

### 2.7. Enzymatic Hydrolysis of FA Nanoprodrugs

The nanoprodrugs were suspended in phosphate buffered saline (PBS, pH 7.4) and esterase (porcine liver, Sigma, code E3019) was added to the final concentration of 5 U/mL. The mixture was incubated for up to 24 h in a water bath at 37°C. To determine the amount of enzymatically hydrolyzed species of the FA prodrugs, the suspensions of nanoprodrugs (100 *μ*L) were added to acetonitrile (300 *μ*L) and analyzed using RP-HPLC as described in Section 2.2.

### 2.8. Intracellular Uptake of Fluorescent-Labeled FA Nanoprodrug in U87 Glioma Cells

To demonstrate intracellular uptake of the nanoprodrugs, cells were incubated in the presence of fluorescent-labeled nanoprodrugs. Four chamber culture slides (BD Biosciences, Bedford, MA) were seeded with U87 cells, and the cells were allowed to attach for 24 h. The medium was replaced with 1.0 mL of freshly prepared suspension of the fluorescent-labeled nanoprodrugs in medium (0.25 *μ*g coumarin 6/mL medium), and the chamber slides were incubated for 5 h. To examine the uptake of free dye, cells were incubated in the dye-treated medium. The dye-treated medium was prepared by incubating the medium in the presence of dye (0.25 *μ*g/mL) for 5 h and sterile filtration. Cells were washed three time with PBS to remove uninternalized nanoprodrugs, one drop of mounting medium with DAPI (Vectashield, Vector Laboratories, Burlingame, CA) was added and then cover slide was placed. For microscopic analysis of intracellular uptake of the fluorescent-labeled nanoprodrugs, a Carl Zeiss Axio Imager Z1 fluorescence microscope equipped with ApoTome (Carl Zeiss MicroImaging, Inc., Thornwood, NY, USA) and Leica DMIRE2 confocal laser-scanning microscope with Confocal Software (Leica Microsystems, Bannockburn, IL, USA) were used. For processing and analysis of the images, AxioVision (Rel. 4.6.3) software (Carl Zeiss) was used. The Carl Zeiss filter with excitation/emission wavelength at 470/525 nm was used.

### 2.9. Cell Counting

U87-MG human glioma cell line was obtained from American Type Culture Collection (ATCC, Bethesda, MD, USA). The cells were grown and maintained as described in [28]. The glioma cells were seeded at 5 × 10^4^ cells per well in 6-well plates containing 2 mL of culture medium and grown for 24 h. The cells were treated with FA nanoprodrugs for 4 days. After treatment, the culture medium was removed and cells were washed with PBS. 0.5 mL of 0.25% Trypsin/EDTA was added to each well and the detached cells were counted immediately in a hemocytometer. The antiproliferative effect of the nanoprodrugs was presented as a cell number % of control, which was calculated as follows:

(2) $$\text{Cell}\;\text{number}\;\text{\%}\;\text{of}\;\text{control}=\left(\frac{\text{Cell}\;{\text{number}}_{\text{treated}}}{\text{Cell}\;{\text{number}}_{\text{control}}}\right)\times 100,$$

where Cell number_treated_ is the number of cells after treatment with nanoprodrugs, and Cell number_control_ is the number of cells of control culture which was incubated with culture medium only. The cells were also treated with control nanosphere prepared from *α*-tocopherol only. The error bar (SD) was calculated from triplicate determinations.

### 2.10. Statistical Analysis

The results were analyzed and expressed as mean ± standard deviation (S.D.). Statistical analysis of the results was carried out using Student's *t*-test. For all tests, differences with a *P* < .05 were considered to be significant.

## 3. Results and Discussion

### 3.1. Preparation of Nanoprodrugs of FA

The synthesis of hydrophobic prodrugs of FA and conversion into nanometer sized prodrugs (nanoprodrugs) offer several advantages which are attributed to the specific characteristics of nanostructures. One of the most remarkable properties of the nanostructured drug and drug delivery system is that a huge surface area is created by transformation of bulk materials into the nanometer-sized. This surface area provides opportunities for chemical and biological interactions between the drugs and biological molecules/enzymes in the physiological environment, leading to an enhanced therapeutic efficacy of the drugs [29, 30]. These properties of nanostructured biomaterials have been routinely exploited for the development of nanostructured prodrugs and drug delivery system.

The increase in hydrophobicity through chemical modification is a crucial factor for the preparation of stable nanostructures using spontaneous emulsification. This is because more hydrophobic compounds can be transformed into more stable nanostructures due to the stronger hydrophobic interaction between the molecules. The resulting hydrophobic nanostructures are stable for a prolonged period of time in an aqueous biological environment, mainly due to the insolubility of the hydrophobically modified compounds and hydrophobic interaction, leading to a strong assembly of the molecules [14]. The hydrophobicity and compact structure may reduce the interaction with water, and thus increase the structural integrity of the nanostructures. 

FA belongs to the acidic NSAIDs that have anti-inflammatory properties linked to COX inhibition [31]. This drug has been reported as an efficient inhibitor of the chlorinating activity of myeloperoxidase (MPO). MPO is a heme-containing enzyme of the peroxidase family that catalyzes the formation of hypochlorous acid (HOCl) in the presence of hydrogen peroxide (H_2_O_2_) and chloride anions (Cl^−^) in the complex defense system against exogenous aggregations [32, 33]. Klabunde et al. showed that FA, along with several NSAIDs and structurally similar compounds, strongly inhibited the formation of insoluble transthyretin (TTR) amyloid fibrils which is known to cause familial amyloid cardiomyopathy and senile systemic amyloidosis [34].

The monomeric derivative FA-TEG-ALA was synthesized using a two-step synthesis as described in Scheme 1(a). TEG was converted to the mono-ALA derivative ALA-TEG-OH, which was followed by the esterification with FA. The secondary aromatic amine in FA did not interfere with the esterification. The dimeric derivative of FA was synthesized using a one-step procedure (Scheme 1(b)). 

**Figure 2:**
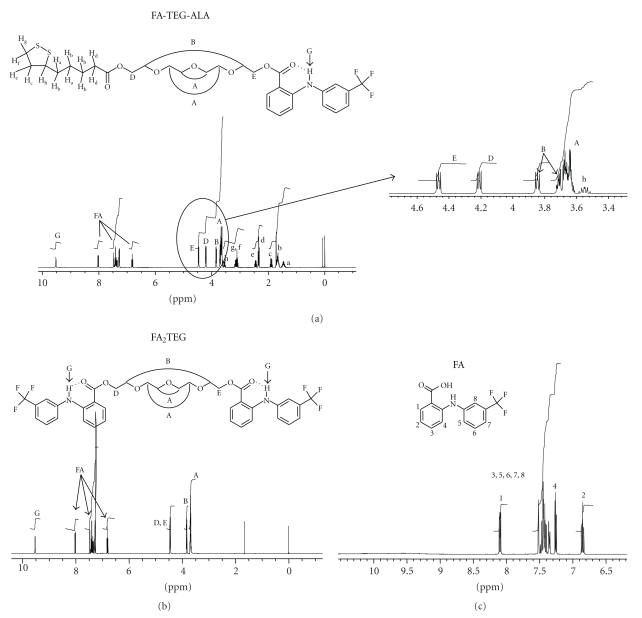
^1^H NMR spectra of (a) FA-TEG-ALA, (b) FA_2_TEG, and (c) flufenamic acid (FA).

The structures were confirmed by ^1^H and ^13^C NMR spectroscopy. The ^1^H NMR data indicate that the resulting spectra are essentially a composite of FA and TEG in the dimeric derivative and a composite of FA, ALA, and TEG in the monomeric derivative (Figure 2). The amine proton in FA is probably involved in a H-bridge with carbonyl oxygen (C=O) as shown in Figure 2. This proton peak at 9.5 ppm was not observed in the spectrum of free FA (Figure 2(c)). The integral of the H-bridged proton was equivalent to one proton in FA-TEG-ALA, while it was equivalent to two protons in FA_2_TEG, reflecting the one and two FA in the FA-TEG-ALA and FA_2_TEG, respectively. The purity of each synthesized compound was analyzed by TLC and RP-HPLC. 

### 3.2. Preparation and Characterization of Nanoprodrugs of FA

The hydrophobic derivatives of FA (Schemes 1(a) and 1(b)) dissolved in acetone spontaneously formed into nanoprodrugs upon the addition into an aqueous solution containing hydrophilic surfactants by spontaneous emulsification process [27, 28, 35–38]. In this study, formulation parameters were kept the same except for the addition of *α*-tocopherol. In the absence of *α*-tocopherol the size of the nanoprodrug prepared from the dimeric FA_2_TEG was significantly smaller than the size the nanoprodrug prepared from the monomeric FA-TEG-ALA, suggesting that a more compact spatial arrangement of the symmetrical dimeric derivative led to the formation of the compacter and smaller nanoprodrug. 

Notably, the retention time of the dimeric FA_2_TEG in RP-HPLC was almost twice as much longer than that of the monomeric FA-TEG-ALA, suggesting a higher hydrophobicity of FA_2_TEG [39, 40]. It can be assumed that the size decreases with increasing hydrophobicity of the compounds, probably due to a stronger hydrophobic interaction between the molecules. In the presence of *α*-tocopherol the size of the FA-TEG-ALA nanoprodrug became significantly smaller when compared with the size in the absence of *α*-tocopherol (Figure 2).

Interestingly, practically no difference in the size was observed for the FA_2_TEG nanoprodrugs in the absence and presence of *α*-tocopherol. This can be explained by the significant increase in the overall hydrophobicity through the addition of *α*-tocopherol in the case of FA-TEG-ALA, whereas it was negligible in the case of FA_2_TEG, probably due to the significant initial hydrophobicity of FA_2_TEG. 

In the presence of *α*-tocopherol the difference in size between the FA_2_TEG and FA-TEG-ALA nanoprodrugs became smaller, which is especially crucial when the therapeutic efficacies of the two nanoprodrugs are to be compared. This is because differences in the therapeutic efficacy can be attributed directly to the different prodrug molecules in the nanoprodrugs when the size and other components do not differ significantly from each other. Thus, the nanoprodrugs were prepared in the presence of *α*-tocopherol for further experiments. 

**Figure 4:**
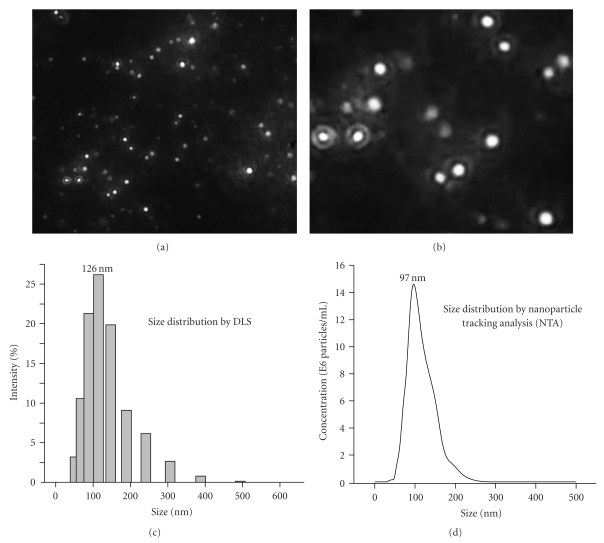
Image of FA-TEG-ALA nanoprodrug obtained from nanoparticle tracking analysis (NTA) (a, b) and size distribution of FA-TEG-ALA nanoprodrug as measured by (c) dynamic light scattering (Coulter N4-Plus Submicron Particle Sizer) and (d) NTA. Image (b) is a magnification of a part of the image (a).

To give a visualization of the nanoprodrugs, we applied the nanoparticle tracking and analysis (NTA) technique which allows direct and real-time visualization of nanoparticles in a liquid as shown in Figures 4(a) and 4(b) [41]. 

Whereas dynamic light scattering (DLS) is an ensemble technique that tries to recover a particle size distribution from the combined signal of all particles present in the sample, nanoparticle tracking analysis (NTA) investigates the diffusion of individual particles. Thus, DLS calculates the average particle diameter by measuring fluctuation in scattering intensity, is highly affected by the presence of a few large particles, and tends to be weighted to the larger particles sizes [42]. Indeed, using DLS (Coulter N4-Plus Submicron Particle Sizer) and NTA for an identical FA-TEG-ALA nanoprodrug, the average size calculated by DLS was 126 nm, which was larger than the size calculated by NTA (97 nm) (Figures 4(c) and 4(d)). The comparison of size distribution and average size from DLS and NTA indicate that few larger nanoprodrugs (>300 nm) have significant influence on the size calculation in DLS. 

**Figure 5:**
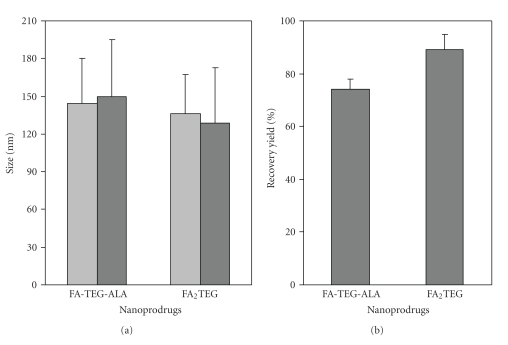
Long-term stability of nanoprodrugs measured by size (a) and recovery yield (b). Light gray bar: before storage; dark gray bar: after 8 weeks of storage. Monomeric nanoprodrug: FA-TEG-ALA; dimeric nanoprodrug: FA_2_TEG. The results are the mean ± SD of three experiments.

The stability of the nanoprodrugs was assessed by measuring the size and contents of the intact FA prodrug molecules after 8-week storage at 4°C. In this study, the size of the nanoprodrugs remained almost unchanged (Figure 5), and the recovery yield of the prodrugs was 75% and 90% for the FA-TEG-ALA and FA_2_TEG, respectively. It is believed that the nanoprodrugs from the more hydrophobic FA_2_TEG formed more stable and compact nanostructures, which can be ascribed to the stronger hydrophobic assembly of FA_2_TEG. This may reduce the interaction with water, and consequently decrease hydrolytic degradation and increase the structural integrity of the nanoprodrug. 

### 3.3. Enzymatic Hydrolysis of FA Nanoprodrugs

**Figure 6:**
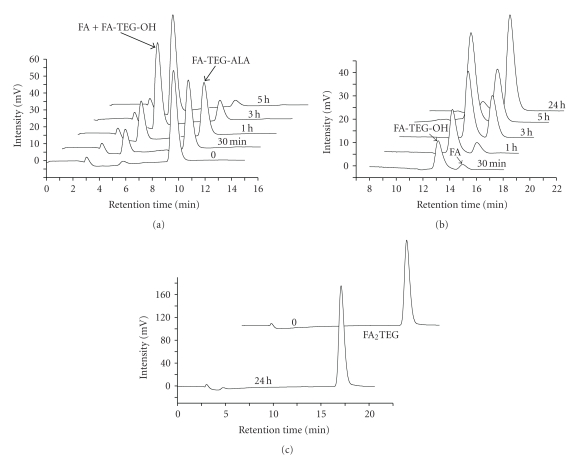
Enzymatic hydrolysis of prodrugs FA-TEG-ALA (a, b) and FA_2_TEG (c) from nanoprodrugs at 37°C. The separation was performed under isocratic condition with a 80/20 (a, c) and 50/50 (b) mixture of acetonitrile/water at a flow rate of 1 mL/min.

**Figure 7:**
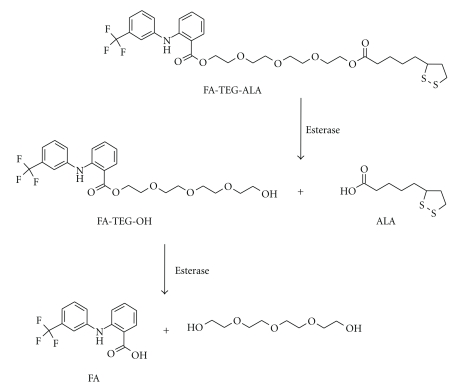
Sequence of enzymatic hydrolysis of FA prodrug FA-TEG-ALA.

In order to assess the enzymatic prodrug activation from the nanoprodrugs, the rate of enzymatic reconversion of the prodrugs into FA and other degradation products was investigated *in vitro* with porcine liver esterase. As shown in Figure 6(a), FA-TEG-ALA nanoprodrug was activated nearly completely after 5 h of incubation at 37°C, whereas no activation was observed in the FA_2_TEG nanoprodrug during the same period of time. This can be attributed to the more hydrophobic nature of the dimeric FA_2_TEG prodrug which makes the interaction between the molecules and enzymes more difficult. In addition, FA is bulkier than ALA, which may increase the steric hindrance towards the enzymes [43, 44]. This assumption was confirmed by the observation that the ALA was first hydrolyzed from FA-TEG-ALA followed by the breakdown of FA-TEG-OH to FA and TEG (Figures 6(c) and 7). 

### 3.4. Intracellular Uptake of Fluorescent-Labeled FA Nanoprodrug in U87 Glioma Cells

**Figure 8:**
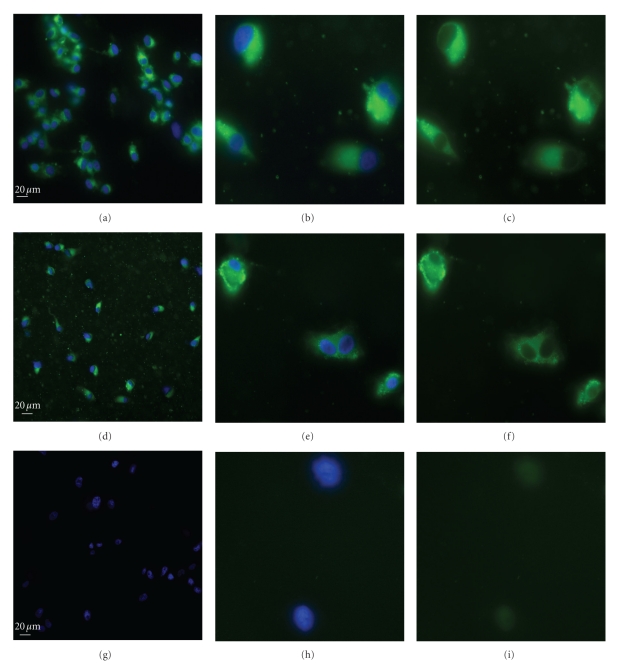
Cellular uptake of fluorescent-labeled nanoprodrugs in U87 glioma cells. Cells were incubated with FA-TEG-ALA nanoprodrug ((a)–(c)), FA_2_TEG nanoprodrug ((d)–(f)) and in dye-treated medium as control ((g)–(i)). Left and middle panels show images of overlapped fluorescence of DAPI and coumarin 6, left with lower and middle with higher magnification. Right panel shows images of fluorescence of coumarin 6 only.

In order to demonstrate the cellular uptake of nanoprodrugs, we prepared fluorescent-labeled nanoprodrugs with the hydrophobic dye, coumarin 6. Due to the hydrophobic nature, the dye remained associated with the nanoprodrugs after overnight dialysis and even after the incubation in PBS buffer and cell culture medium [45]. Confocal laser scanning microscopy of U87 glioma cells treated with fluorescent-labeled nanoprodrugs showed strong internalization of the nanoprodrugs within 5 h of incubation. Both nanoprodrugs FA-TEG-ALA (Figures 8(a)–8(c)) and FA_2_TEG (Figures 8(d)–8(f)) showed similar cellular uptake, whereas cells incubated in the dye-treated control medium did not show any detectable fluorescence (Figures 8(g)–8(i)).

Some cells showed a stronger accumulation along the membrane area, while other showed more evenly distributed pattern in the cytoplasm. Interestingly, cells contained numerous tiny vesicles that were dispersed in the cytoplasmic compartment. The vesicles are probably endosomal vesicles (endosomes), suggesting that the cellular uptake occurs via endocytosis. Considering the different spatial intensity and localization of the fluorescent signals within the cells, it can be concluded that after endocytosis the nanoprodrugs escape from the endosomes to the cytoplasm and are dispersed evenly throughout the cytoplasm. 

### 3.5. Effect of FA Nanoprodrug on Cell Proliferation

**Figure 9:**
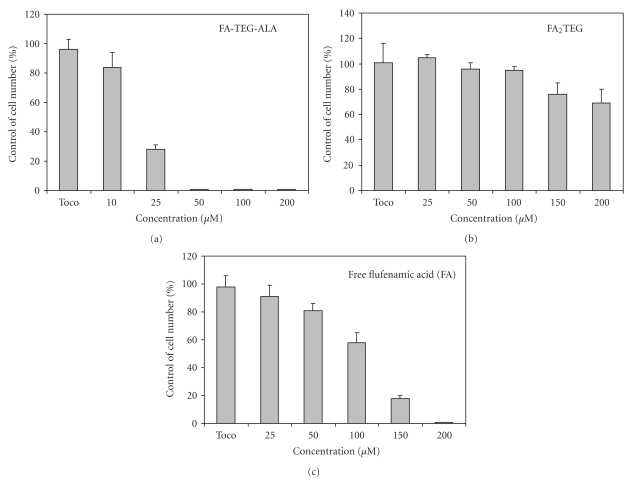
Effect of nanoprodrug of FA-TEG-ALA (a), nanoprodrug of FA_2_TEG (b), and free flufenamic acid (c) on the viability of U87-MG glioma cells.

In order to evaluate the effect of FA nanoprodrugs on tumor cell growth, we studied the effect on the cell growth of U87-MG glioma cells. Glioma cells were treated with nanoprodrugs from FA-TEG-ALA and FA_2_TEG, and also with FA in the concentration range of 10 to 200 *μ*M. Cells were also treated with control nanospheres prepared from *α*-tocopherol only by exposure to an equimolar concentration of *α*-tocopherol. As shown in Figure 6, the nanoprodrug of FA-TEG-ALA completely inhibited the cell proliferation at the concentration of 50 *μ*M, whereas the nanoprodrug of FA_2_TEG inhibited only 30% at the highest concentration of 200 *μ*M. These results were well expected because the prodrug FA_2_TEG was almost inert towards chemical and enzymatic hydrolysis (Sections 3.2 and 3.3). Based on the results of this study, the stability and biodegradability of the nanoprodrugs may be adjusted to meet the needs for diverse practical applications via modification of prodrug structures. Interestingly, the inhibitory effect of the nanoprodrug FA-TEG-ALA was much higher than that of the FA (Figure 9(c)), suggesting the existence of more efficient cellular delivery mechanism for the nanoprodrug.

## 4. Conclusion

In this study we showed that hydrophobic derivative of FA can be formed into stable nanoprodrug that is readily activated by hydrolytic enzyme and inhibits the growth of malignant cells, suggesting their potential application as anticancer agents. Nanoprodrugs of FA were prepared by spontaneous emulsification of the monomeric prodrug FA-TEG-ALA and dimeric FA_2_TEG, and their antiproliferative effects were demonstrated using U87-MG glioma cells. The nanoprodrug from FA-TEG-ALA inhibited the cell growth significantly and induced cell death, whereas the nanoprodrug from FA_2_TEG did not show any comparable effect on cell growth and viability. We demonstrated using fluorescent-labeded nanoprodrugs that both nanoprodrugs FA-TEG-ALA and FA_2_TEG showed similar cellular uptake. Obviously, the more potent effect of the monomeric nanoprodrug is due to the higher parent drug concentration which is ascribed to the higher enzymatic activation. In addition, the FA-TEG-ALA nanoprodrug inhibited cell growth more efficiently than free FA, suggesting a delivery mechanism specific to the nanoprodrug. We are currently investigating the mechanisms of the cellular uptake and the molecular events leading to the antiproliferative effect of the FA nanoprodrug.

## Figures and Tables

**Figure 3:**
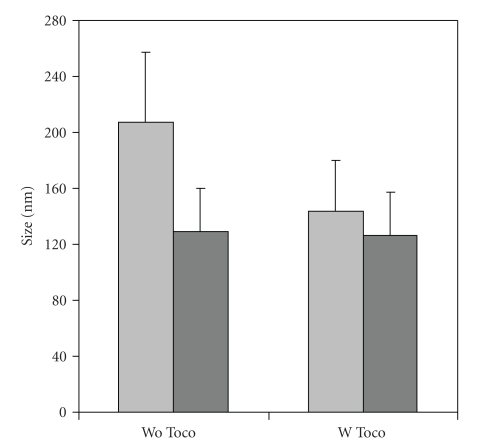
Size of the nanoprodrugs in the absence (wo Toco) and presence (w Toco) of *α*-tocopherol. Light gray bar: monomeric FA-TEG-ALA; dark gray bar: dimeric FA_2_TEG.
